# Scoring method of English composition integrating deep learning in higher vocational colleges

**DOI:** 10.1038/s41598-024-57419-x

**Published:** 2024-03-27

**Authors:** Shuo Feng, Lixia Yu, Fen Liu

**Affiliations:** Department of Culture, Sports and Labor, Gannan Healthcare Vocational College, Ganzhou, 341000 China

**Keywords:** Deep learning, Higher vocational colleges, Natural language processing, English composition, Intelligent scoring, Psychology, Engineering

## Abstract

Along with the progress of natural language processing technology and deep learning, the subjectivity, slow feedback, and long grading time of traditional English essay grading have been addressed. Intelligent English automatic scoring has been widely concerned by scholars. Given the limitations of topic relevance feature extraction methods and traditional automatic grading methods for English compositions, a topic decision model is proposed to calculate the topic relevance score of the topic richness in English composition. Then, based on the Score of Relevance Based on Topic Richness (TRSR) calculation method, an intelligent English composition scoring method combining artificial feature extraction and deep learning is designed. From the findings, the Topic Decision (TD) model achieved the best effect only when it was iterated 80 times. The corresponding accuracy, recall and F1 value were 0.97, 0.93 and 0.95 respectively. The model training loss finally stabilized at 0.03. The Intelligent English Composition Grading Method Integrating Deep Learning (DLIECG) method has the best overall performance and the best performance on dataset P. To sum up, the intelligent English composition scoring method has better effectiveness and reliability.

## Introduction

Language is a bridge between international communication and academic research. English is also a compulsory course in compulsory education and higher vocational colleges in China. However, in China, English exam-oriented education focuses on the written tests, which comprehensively evaluate students' English proficiency based on their vocabulary proficiency, reading comprehension ability, and English writing ability. Among them, the English composition exam is a comprehensive examination of students' language ability from words, grammar, long and difficult sentences and overall text expression ability. The traditional offline English teaching is widely used in higher vocational colleges. There is often a serious contradiction between effective classroom teaching practice and many students. It is hard for teachers to check every student efficiently and comprehensively. It is also difficult to provide timely feedback on students' writing issues. In addition, the subjective factors of teachers also affect composition judgment. According to the statistics of the Ministry of Education, the students in China have displayed a significant upward trend, but the number of English teachers has declined ^1^. In this context, English teaching has become a heavy burden for teachers. It is also difficult for students to receive timely feedback from teachers on composition problems. Liu H et al. proposed a new relationship-driven method based on Transformer architecture. A new token-guided multiple loss function was designed to solve the severe occlusion, low illumination and extreme direction existing in head pose estimation in practical applications. Based on the experimental results of three challenging benchmark HPE datasets, the proposed approach achieved state-of-the-art performance ^2^. Liu et al., proposed a human pose estimation model with joint direction cue and Gaussian coordinate coding to alleviate the constraints of human pose estimation under normal circumstances. Experimental results showed that this method could obtain robust results. The extended experiments were carried out on the collected infrared images. The results indicate that the experiment achieved good results when there was insufficient color and texture information ^3^. Liu et al., designed an efficient deep matrix decomposition with retrospective feature learning for industrial recommendation systems to explain the characteristics of user reviews. The research results on multiple data sets showed that the proposed method was superior to existing methods in terms of effectiveness and efficiency. It had a good prospect for industrial transformation and application ^4^. Therefore, it is the most important thing to realize the intelligent scoring method of English composition with Internet technology. With the gradual maturity of Internet technology, it is possible to combine it with education. The Score of Relevance Based on Topic Richness (TRSR) is designed to address the dimension of topic richness in English compositions, aiming to achieve objective and efficient intelligent scoring. Combined with Deep Learning ^5–7^, an Intelligent English Composition Grading Method Integrating Deep Learning (DLIECG) is proposed.

## Related work

In recent years, artificial intelligence has been widely applied in various fields. The automatic grading of English compositions has also received extensive attention from researchers ^8^. Automatic grading of English composition is to solve the heavy teaching burden, strong subjectivity of composition grading, long examination time and difficult feedback in traditional English teaching ^9^. Many scholars have conducted in-depth analysis and discussion. Rajagede designed a model for automatically evaluating student essay answers for automatic grading of Indonesian student essays. The results showed that the model extracted more information from sentences. However, the file size was smaller than the Fast-Text pre-training model. On the Ukara dataset, the model had a better F1 value, at 0.829 ^10^. Under the background of computer technology and AI technology, Yi found that automatic English grading was the inevitable trend. He proposed a college English assistance system solution based on artificial intelligence technology. This paper discussed the application of AI in English teaching to improve the English teaching effect ^11^. Ince et al., aimed to develop an objective and effective automatic scoring model for open questions using machine learning method. The research results showed that this method had the best precision and F1 value in the Türkiye physics curriculum dataset ^12^.

The deep learning has achieved relatively mature application achievements. The composition scoring method based on deep learning can solve the difficult semantic information extraction relying on artificial features, maintaining excellent performance in most tasks ^13^. However, in the practical application of English composition grading, due to the constraints of scale, the generalization ability of the model is defective, resulting in the inability to recognize the shallow features ^14^. Wang et al., found that traditional machine learning couldn’t be directly applied. Therefore, deep learning was introduced to design an English word segmentation processing method with multiple neural networks. The experimental results showed that the average prediction processing speed of this method was 1.94 times faster than BI-LSTM-CRF, indicating that the proposed method had a faster processing speed. It could effectively improve the efficiency of word segmentation processing ^15^. Cui analyzed the application of deep learning and object visual detection in online English vocabulary teaching. The results showed that the application of corpora in university vocabulary teaching could promote students to actively use corpora in English vocabulary learning. The classification accuracy of this method was over 90% ^16^. Hao found that students didn’t have sufficient interactive interest and emotional stimulation in multimedia English teaching. For this defect, an intelligent network English teaching system based on deep learning speech enhancement and facial expression recognition was studied. The experimental results confirmed that it had good detection ability on students' expressions ^17^.

To sum up, the research on automatic English grading methods is not mature, but it can learn from other automatic essay grading systems. In view of the lack of relevance dimension research in the existing English scoring system and the feature deficiencies in deep learning, the research first proposes a TRSR model based on topic richness. Then, deep learning is combined with artificial feature extraction methods to construct the DLIECG method.

## Design of intelligent English composition scoring method integrating deep learning

### Preparation stage of intelligent English composition scoring method

**Figure 1:**
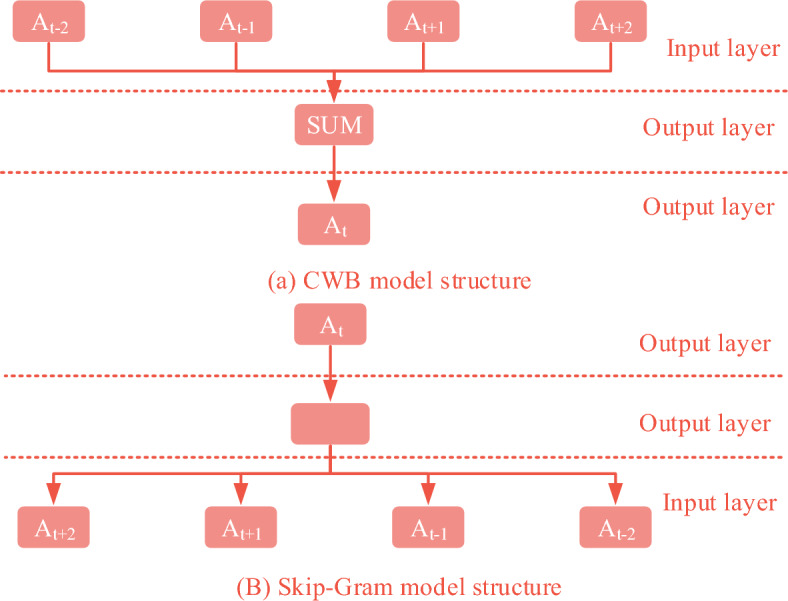
Structure diagram of CWB model and skip-gram model.

The preparation stage of intelligent English composition scoring method includes Pre-training Word Vector (PWV), Recurrent Neural Network (RNN), Transfer Learning (TL) and Text Segmentation (TS) ^18–20^. PWV encodes syntactic and semantic information into a dense vector, which solves the dimensionality curse caused by traditional single hot encoding. Among them, single hot encoding mainly encodes N states through N-bit state registers. Each state is represented by a corresponding independent register bit, which is only valid for one bit at any time. The dimensionality curse problem is that a single hot encoding introduces a large number of new features based on the original features, leading to dimension explosion. Especially in situations with multiple classifications, this may increase computational complexity and storage space requirements. At present, the mainstream word vector construction work includes the Context-based Pre-training Word Vector Construction method (Word2vec), the Global Vectors for Word Representation (Glove) and the Transformer-based Pre-training model (BERT) ^21,22^. In the Word2vec model training, the Continuous Word Bag model (CWB) for predicting intermediate words in the sliding window and the Skip-Gram model for predicting two words on both sides of the known intermediate words are shown in Fig. 1.

In Fig. 1a, the CWB model is composed of input layer, projection layer and output layer. $\left[ {A_{t - 2} ,A_{t - 1} ,A_{t} ,A_{t + 1} ,A_{t + 2} ,} \right]$ is the current window contains words. The window word is first thermally coded separately as the input layer. The coding dimension is the non-repeating thesaurus set of the current corpus. The unique heat code of $\left[ {A_{t - 2} ,A_{t - 1} ,A_{t + 1} ,A_{t + 2} ,} \right]$ is accumulated and summed on the projection layer. Secondly, the summation coding is used as the input layer. The SoftMax function is used to classify and predict the prediction words. Finally, the network parameters are optimized by back-propagation algorithm. CWB uses a Huffman tree to classify the output layer for optimized computation. In Fig. 1b, the speed of the skip-Gram model training word vector is opposite to that of CWB. The input is the unique code of the target word, and the output is the words on both sides of the window. The core task of the Skip-Gram model is to learn a mapping relationship that maps words into a vector space, so that semantically similar words have close distances in the vector space. Glove uses the property of $ratio$ to establish the loss function by connecting with the word vector. The least square loss is optimized using the Adarad method. The construction process of co-occurrence matrix is as follows. $U$ is the co-occurrence matrix. The element is $U_{j,k}$, which represents the number of times that the words $j$ and $k$ appear together in a window. $B = \left\{ {Anny,I,like,you,but,you,like,her} \right\}$ is the corpus. The vocabulary size is $N = 6$. Assuming that the current sliding window width is 5, a window content will be generated after one sliding. Taking Window 3 as an example, that is, the head word is you, and the context words are I, like, but and you. The formula (1) can be obtained.

1 $$\left\{ \begin{gathered} U_{you,i} + = 1 \hfill \\ U_{you,like} + = 1 \hfill \\ U_{you,but} + = 1 \hfill \\ U_{you,you} + = 1 \hfill \\ \end{gathered} \right.$$

Taking formula (1) as an example, the co-occurrence matrix $U_{6 \times 6}$ is obtained by sliding $B$. The number of times two adjacent words appeared together in $B$ is stored by co-occurrence matrix. Then the word vector is constructed through $ratio$ feature, as shown in formula (2).

2 $$\left\{ \begin{gathered} ratio_{i,j,k} = \frac{{P_{i,k} }}{{P_{j,k} }} = \frac{{\exp \left( {b_{i}^{T} b_{k} } \right)}}{{\exp \left( {b_{j}^{T} b_{k} } \right)}} \hfill \\ P_{i,k} = \frac{{U_{i,k} }}{{U_{i} }} \hfill \\ U_{i} = \sum\limits_{j = 1}^{N} {U_{i,j} } \hfill \\ \end{gathered} \right.$$

In formula (2), $P_{i,k}$ and $P_{i,k}$ represent the occurrences of the word $k$ in the context of $i$ and $j$. $b_{i}$, $b_{j}$ and $b_{k}$ are vector representations of the current words $i$, $j$ and $k$. $U_{i,k}$ is the number of occurrences of the word $k$ in the $i$ context. $U_{i}$ represents the number of occurrences of the word $i$. Table 1 shows the property of $ratio$.

**Table 1:** $ratio$ value property.

The value of $ratio_{i,j,k}$	Words $j$ and $k$ are related	Words $j$ and $k$ are unrelated
Words $i$ and $k$ are related	Close to 1	Very big
Words $i$ and $k$ are unrelated	Very small	Close to 1

**Figure 2:**
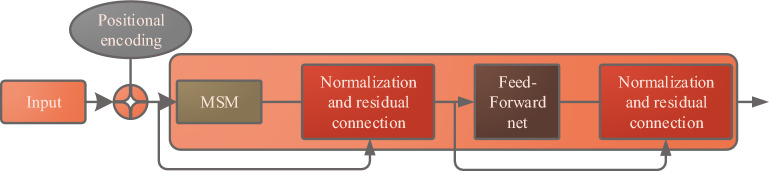
Transformer encoder unit structure.

BERT model is universal. Based on the transformer encoder part, it uses the Masked Language Model (MLM) and the Next Sentence Prediction (NSP) training task to train on the data. The structure of Transformer encoder unit is shown in Fig. 2.

In Fig. 2, the unit is composed of a Multi-headed Self-attention Mechanism (MSM) and a fully connected forward propagation network. Normalization and residual connection are introduced in this unit. The MSM allocates the weight of input codes, accumulates and transmits the codes after each Attention Mechanism (AM) to the forward network. After the forward network normalizes the coding, it can be output to the next transformer encoder unit by adding the coding before transmission. BERT model uses the processing mode of transformer encoder to encode the bidirectional context information based on the MLM pre-training task. RNN is widely used in Natural Language Processing (NLP), which solves the unordered input information in feedforward neural networks, large space occupied by traditional language models, and the inability of convolutional neural networks to extract global semantics. RNN can capture the information that has been calculated in the history for calculating the current time. At present, the mainstream RNN-based method is the Long Short-term Memory Network (LSTM). The model optimizes the structure of RNN, adds $Cell$ state unit, and alleviates the gradient disappearance problem caused by RNN structure. $Cell$ structure is composed of input gate $s$, forgetting gate $f$ and output gate . The relevant calculation is shown in formula (3).

3 $$\left\{ \begin{gathered} s_{t} = \delta \left( {\omega_{s} \cdot \left[ {h_{t - 1} ,u_{t} } \right] + d_{s} } \right) \hfill \\ f_{t} = \delta \left( {\omega_{f} \cdot \left[ {h_{t - 1} ,u_{t} } \right] + d_{f} } \right) \hfill \\ o_{t} = \delta \left( {\omega_{o} \cdot \left[ {h_{t - 1} ,u_{t} } \right] + d_{o} } \right) \hfill \\ \end{gathered} \right.$$

In formula (3), $\delta$ is the activation function. $\omega$ is the weight matrix. $$\left\{ \begin{gathered} CL_{t} \hfill \\ CL_{t} \hfill \\ h_{t} \hfill \\ \end{gathered} \right.$$ is the deviation value. The calculation of $Cell$ unit $CL_{t}$ and hidden layer state $h_{t}$ is shown in formula (4).

4 $$\left\{ \begin{gathered} C\tilde{L}_{t} = \tanh \left( {\omega_{CL} \cdot \left[ {h_{t - 1} ,u_{t} } \right] + d_{CL} } \right) \hfill \\ CL_{t} = f_{t} \cdot CL_{t - 1} + \left( {1 - f_{t} } \right) \cdot C\tilde{L}_{t} \hfill \\ h_{t} = o_{t} \cdot \tanh \left( {CL_{t} } \right) \hfill \\ \end{gathered} \right.$$

The deep learning or machine learning is mainly driven by supervised learning. Supervised learning needs to rely on abundant labeled data to train a successful model, which also reflects the shortcomings of deep learning and machine learning. TL can solve the contradiction between big data and few labels, as well as between general models and personalized needs. TL can be divided into TL methods based on features, instances, relationships and models according to learning methods. The model-based TL method trains the model in the source domain through a large number of data. It is used for the process prediction in the target domain. Written text is separated into meaningful units through TS. According to the granularity of segmentation, there are basic discourse unit segmentation tasks and topic segmentation tasks. Among them, topic segmentation is to divide a section of text through topic semantic information, with each topic being continuous.

### Calculation method of TRSR and design of DLIECG

**Figure 3:**
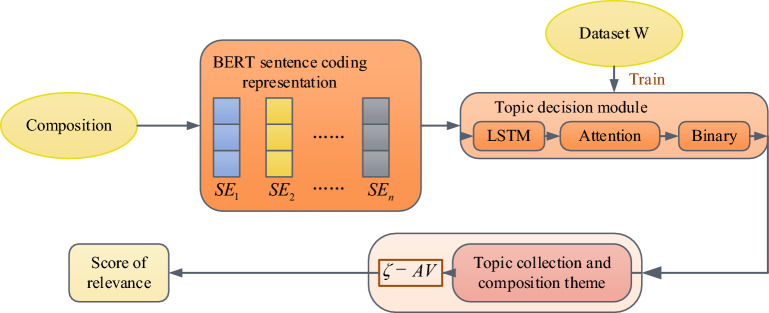
TRSR calculation method flow.

At present, the automatic analysis system for English composition in the education application market has the following problems, such as the imperfect feedback mechanism and the lack of feedback on relevance and other dimensions. Nowadays, the mainstream correlation methods in English composition include deep learning and unsupervised feature extraction. These two methods lack the fine-grained analysis of content and face difficulties in extracting semantic features. However, the unsupervised method can effectively avoid the defect of relying on annotation data. The corresponding semantic information can be extracted by deep learning method. Unsupervised feature extraction methods often treat the task as a semantic similarity problem. Firstly, feature selection methods are used to extract text features of an essay, such as keywords and topic features. Then, based on the extracted features, each text is transformed into a vector. Finally, the relevance of the essay is determined by calculating the similarity between the text vector and the essay topic vector. At present, the NLP is conducted in a pre-trained model environment, which makes it possible to obtain better sentence semantic vector representation through the pre-training model. The study combines the advantages of feature extraction methods with the pre-trained model in semantic vectorization representation. A TRSR calculation method is proposed to optimize the feedback mechanism of an intelligent English essay scoring system. Among them, topic richness is the correlation between the number of topics and the requirements of an English essay. The correlation is semantic similarity. The TRSR calculation method is shown in Fig. 3.

In Fig. 3, the specific flow of TRSR calculation method is as follows. The first step is to code the acquired English composition data and input it into the model. The second step is to obtain relevant data with topic granularity through the model. The third step is to quantify the theme and its semantics in English composition. The fourth step adopts $\zeta - AV$ to calculation and get the final score of the the English composition correlation. The bidirectional coding BERT model based on transformer can learn semantic features at high level, which can make the text data obtain better representation. Therefore, the input of Topic Decision model (TD) uses the bidirectional coding method of BERT model. The sentence is expressed as $SE = \left( {l_{1} ,l_{2} , \ldots ,l_{n} } \right)$. $l_{j} ,1 \le j \le n$ is the $j$ word of the current sentence. The word vector $v_{j}$ is obtained through the BERT pre-training model for all words. The sentence coding method uses the average value of 9–12 layers of coding in the model to represent $v_{j}$, as shown in formula (5).

5 $$v_{j} = BERT^{RT} \left( {l_{j} } \right)$$

In formula (5), $RT$ represents the specific coding method of $v_{j}$. The $RT$ is shown in formula (6).

6 $$RT = \frac{{\sum\limits_{m} {layer_{m} } }}{3}$$

In formula (6), $layer_{m}$ is the BERT code of layer $m$. The research uses TD model to segment the topic granularity of English composition data. The last sentence of each topic in the text is taken as the stop point, and the input sentence is determined using the structure of BILSTM-Attention model. In the TD model, the first step is to input the word granularity. Then the AM is used to give the input data weight, and a fully connected layer with dimensionality reduction is input. Finally, the SoftMax function is used to classify. Sentences $SE_{j} ,SE_{j + 1} = \left[ {v_{1} ,v_{2} , \cdots ,v_{q} } \right]$ are entered. The BiLSTM model codes input data, as shown in formula (7).

7 $$b_{t} = BiLSTM\left( {v_{t} } \right)$$

In formula (7), $b_{t} \left( {1 \le t \le q} \right)$ represents the encoding of $v_{t}$ by BiLSTM at $t$. Then AM is used to calculate the output weight of BiLSTM at each time point. Formula (8) displays the process.

8 $$\left\{ \begin{gathered} x_{t} = \tanh \left( {\omega_{v} \cdot b_{t} + d_{v} } \right) \hfill \\ e_{t} = \frac{{\exp \left( {x_{t}^{T} \cdot x_{v} } \right)}}{{\sum\limits_{t} {\exp \left( {x_{t}^{T} \cdot x_{v} } \right)} }} \hfill \\ \end{gathered} \right.$$

In formula (8), $x_{t}$, $\omega_{v}$ and $d_{v}$ represent the number of layers of AM. $e_{t}$ means that the input sequence of the $t$ time point accounts for the weight of all inputs. The input vector $y_{t}$ with weight expression can be obtained in the AM layer, as shown in formula (9).

9 $$y_{t} = e_{t} \cdot b_{t}$$

The vector representation with vocabulary weight is calculated by AM. It is entered into the full connection layer. To realize classification, the SoftMax function is adopted. The vector after splicing is $y^{\prime}$, as shown in formula (10).

10 $$\left\{ \begin{gathered} y = con\left( {y_{1} ,y_{2} , \ldots ,y_{q} } \right) \hfill \\ y^{\prime} = fc\left( y \right) \hfill \\ \tilde{z} = soft\max \left( {W_{s} \cdot y^{\prime} + d_{s} } \right) \hfill \\ \end{gathered} \right.$$

In formula (10), $con$ represents the splicing of vectors. $W$ and $d_{s}$ are network parameters of the current classification layer. $\tilde{z}$ is the final classification outcome. The cross entropy loss function is the loss function of TD model. The current training batch $G = \left( {g_{1} ,g_{2} , \ldots ,g_{n} } \right)$ of $n$ training samples is added. The Mean Square Error (MSE) function is shown in formula (11).

11 $$loss\left( {Z_{G} ,Z_{{\tilde{G}}} } \right) = - \frac{1}{N}\sum\limits_{j = 1}^{n} {\left( {z_{j} \cdot \log \left( {\tilde{z}_{j} } \right) + \left( {1 - z_{j} } \right) \cdot \log \left( {1 - \tilde{z}_{j} } \right)} \right)}$$

In formula (11), $Z_{G}$ and $Z_{{\tilde{G}}}$ represent the data batches of actual category and forecast category, respectively. The key is to obtain the similarity between topics and semantic vectorization of English compositions. The BERT-Sentence model solves the problem that the traditional BERT model takes a large part in calculating the semantic similarity of sentences. BERT-Flow model presents non-smooth anisotropy to BERT's semantic space, which optimizes the semantic space distribution. Therefore, BERT-Sentence model and BERT-Flow model are selected for data semantic vectorization. Semantic expression ability $\zeta$ of BERY model and Fast-Text are tested. When calculating the $\zeta - AV$ score, $\zeta$ is the reward factor. $AV$ is the mean value idea. The split English composition data is $EE = \left\{ {tc_{1} ,tc_{2} , \ldots ,tc_{n} } \right\}$, which includes $n$ topics. $tc_{j} \left( {1 \le j \le n} \right)$ stands for the $j$ topic in the composition. Semantic vectorization and English composition theme are $tc$ and $te$. The semantic similarity of each $tc$ vector and $te$ vector are calculated to get the relevance degree $S$, as shown in formula (12).

12 $$S = \frac{1}{n + 2}\left( {\left( {\sum\limits_{j = 1}^{n} {SI^{j} } } \right) + \zeta + SI^{EE} } \right)$$

**Figure 4:**
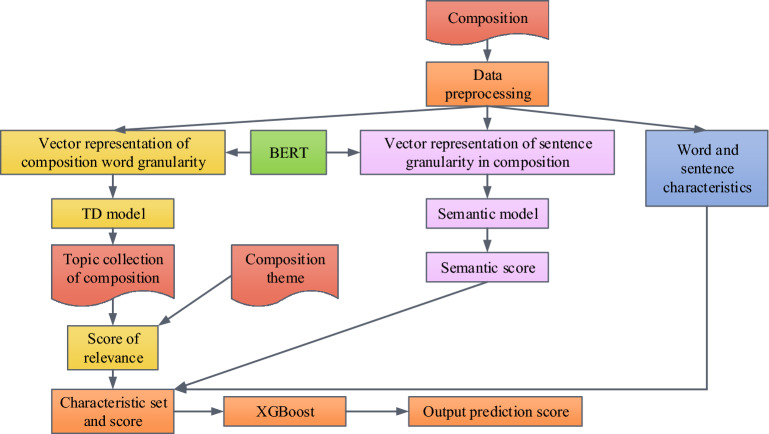
DLIECG method flow.

In formula (12), $SI^{j}$ is the similarity between $tc$ and $te$. $SI^{EE}$ represents the similarity between the whole English composition and $te$. A higher $\zeta$ value indicates more $tc$ in English composition. $\zeta$ will gradually approach 1 with the growth of $tc$ to reduce the influence of extreme topics in English composition. By combining the advantages of artificial features and semantic scoring models to extract feature points, as well as the TRSR calculation method, an enhanced deep learning IECG method can be obtained. Figure 4 presents the specific process.

The artificial feature method is applied to extract shallow features. Feature extraction is performed on students' vocabulary and sentence abilities. Table 2 shows the details.

**Table 2:** Features at word and sentence level.

Characteristic level	Characteristic name	Characteristic description
Word	Word length variance	Word length variance
	The ratio of the number of sentences to the number of words	The ratio of the number of sentences to the number of words
	Number of words	Total number of words in the whole composition
	Average word length	Average number of characters per word
	Proportion of vocabulary in CET4 and CET6	The ratio of words in CET-4 and CET-6 to the total number of words
	The ratio between the number of connectives and the use of prepositions	The ratio of the number of conjunctions and prepositions to the total number of words
	Misspelled words	Number of misspelled words
Sentence	Sentence readability	The weighted sum of the average number of characters in a word and the average length of a sentence
	Average sentence length	Average sentence length
	Composition length	Total number of sentences in the composition
	Number of sentence grammatical errors	Number of grammatical errors with sentence granularity

The research uses the Bi-directional LSTM model (BiLSTM) to build a model for the semantic score $EE$ of English compositions. The first is the vector representation of model input. The current English composition is $EE = \left\{ {SE_{1} ,SE_{2} , \ldots ,SE_{n} } \right\}$. $EE$ is composed of $n$ sentences $SE$. The specific code of $SE$ is shown in formula (13).

13 $$SE = \frac{1}{n}\sum\limits_{i}^{n} {l_{i} }$$

To gain the context semantic information, the research uses the BiLSTM model. Compared with the traditional LSTM, the output information of the BiLSTM model at $t$ is $h_{t}$. This model not only extracts the information of the first $t - 1$ time, but also fuses the information after the $t$ time. If $EE = \left\{ {SE_{1} , \ldots ,SE_{t - 1} ,SE_{t} ,SE_{t + 1} , \ldots ,SE_{n} } \right\}$, the encoded representation of BiLSTM can be obtained, as shown in formula (14).

14 $$\left\{ \begin{gathered} \mathop{h}\limits^{\leftarrow} _{t} = LSTM\left( {SE_{1} , \ldots ,SE_{t - 1} ,SE_{t} ,SE_{t + 1} , \ldots ,SE_{n} } \right) \hfill \\ \vec{h}_{t} = LSTM\left( {SE_{1} , \ldots ,SE_{t - 1} ,SE_{t} ,SE_{t + 1} , \ldots ,SE_{n} } \right) \hfill \\ \end{gathered} \right.$$

In formula (14), $\mathop{h}\limits^{\leftarrow} _{t}$ and $\vec{h}_{t}$ represent the forward and reverse outputs of the BiLSTM model, respectively. The final output is the forward and reverse output vector splicing to represent $H$. The dimension of $H$ is reduced by full connection layer. The vector after dimension reduction is expressed as $H^{\prime}$. The activation function sigmoid obtains the score of [0,1] by formula (15).

15 $$Z^{\prime}_{G} = \delta \left( {H^{\prime}} \right)$$

In formula (15), $Z^{\prime}_{G}$ is the semantic score of current $Z^{\prime}_{G}$. Another loss function of the model is MSE function. If there are $n$ training samples and the training batch is $R = \left\{ {EE_{1} ,EE_{2} , \ldots ,EE_{n} } \right\}$, the loss function is calculated, as shown in formula (16).

16 $$Loss\left( {Z_{G} ,Z^{\prime}_{G} } \right) = \frac{1}{N}\left( {Z_{G} - Z^{\prime}_{G} } \right)^{2}$$

In formula (16), $Z_{G}$ represents the total score of English composition in the real dataset. $Z^{\prime}_{G}$ represents the predicted semantic score.

## Performance analysis of intelligent English composition scoring method integrated with deep learning

To prove the classification effect of the TD proposed in the study, the accuracy rate, recall rate and F1 value are used as evaluation indicators. The study uses data from datasets P and W. There are five groups of compositions in dataset P, each of which contains one topic, all of which are completed by students in the same year of higher vocational colleges. Each composition has a corresponding score. Dataset W contains 700,000 documents from the English Wikipedia and filters data with a data size of 25 sentences. The data of dataset P and dataset W are divided. The ratio of training, testing and verification is 8:1:1. The training set is used to better learn the features and patterns of the task. The verification set is used to adjust the hyper-parameters of the model and monitor whether the model is over fitting or under fitting. The testing set is used to evaluate the performance of the trained model on the dataset. The input dimension parameters, hidden layer size and model depth of TD model are 768, 64 and 2, respectively. The random number seed is 42.

**Figure 5:**
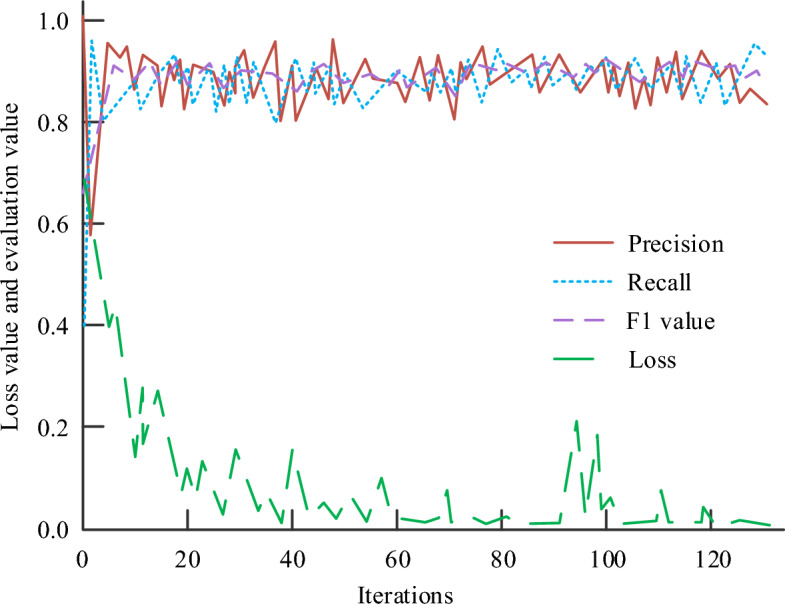
Training results of TD model.

After the TD model is trained, the iterative change curves of model loss, accuracy, recall rate and F1 value on the test set are shown in Fig. 5. From Fig. 5, the best effect was achieved when the model was iterated to 80 times. The accuracy, recall and F1 value were 0.97, 0.93, and 0.95. The model training loss value started to stabilize and finally stabilized at 0.03. The research results show that TD algorithm can well learn the irrelevant information between the truncation point and the first sentence of the new topic. It can determine whether the sentence is the segmentation point of the composition topic and whether the two sentences belong to the same topic.

**Figure 6:**
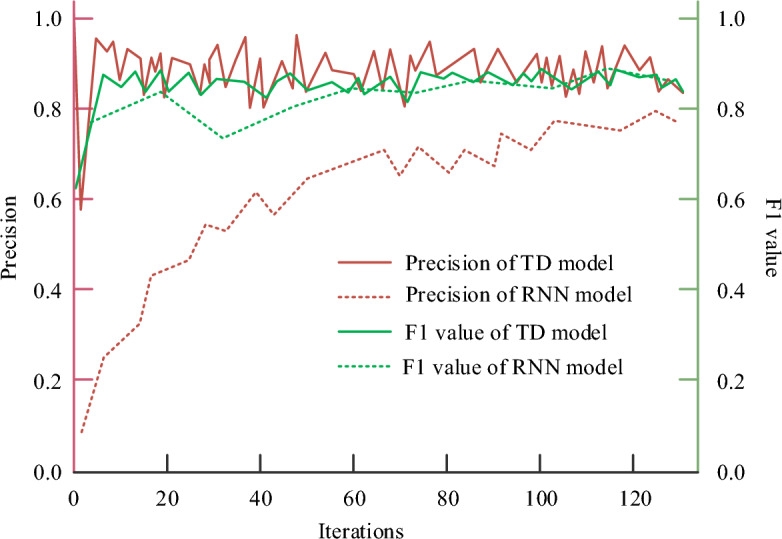
Accuracy and F1 value results of different models.

To further scientifically verify the performance of TD model, RNN model is selected for comparison. The accuracy of different models is compared with the F1 value training results. From Fig. 6, the accuracy and F1 value proposed in the study were the highest, with 97.83% and 95.36% respectively. The curve fluctuated slightly. The accuracy and F1 value of RNN model were low, with 92.16% and 90.67% respectively, and the curve fluctuated greatly. It indicates that the performance of RNN model is unstable, while the TD has higher accuracy.

To prove the validity of the TRSR proposed in the study, the study conducts an experiment on dataset P. Then the Pearson Coefficient (Per) is used to evaluate the correlation between the relevance score and the total score of English composition. A high Per value indicates a strong correlation between the correlation score and the total score of English composition. After testing the dataset P, the Per results under different vectorization methods can be obtained, as shown in Table 3. The Per value was affected by the students’ grade, the difficulty of the composition theme and other variables. Students’ grades and compositions were integrated. Therefore, the control variable method is used to analyze the results in Table 3. From Table 3, under the same composition collection, the Per value and relevance score obtained by different semantic vectorization methods were different. The Per value under the BERT and Fast-Text vector methods did not conform to the results under the influence of the interaction between variables. The theme difficulty of composition collections 1 and 2 is the same. The academic year is the first and third year of higher vocational education. In theory, the writing ability of the third grade students in higher vocational colleges should be better than that of the first grade students, but the Per value performance of BERT method was contrary to the other three methods. BERT-Sentence and BERT-Flow methods could comprehensively reflect the accurate Per value. For composition collection 3 with a difficult topic, it showed a low Per value. To sum up, experiments on dataset P using different semantic vectorization methods have verified that the TRSR calculation method proposed in the study conforms to the results under the comprehensive influence of multiple variables.

**Table 3:** Per value results under different vectorization methods.

Composition collection	1	2	3	4	5
BERT	0.156	0.132	0.413	0.151	0.561
BERT-Sentence	0.540	0.561	0.160	0.256	0.304
Fast-Text	0.133	0.242	0.406	0.107	0.521
BERT-Flow	0.553	0.572	0.233	0.285	0.342

**Figure 7:**
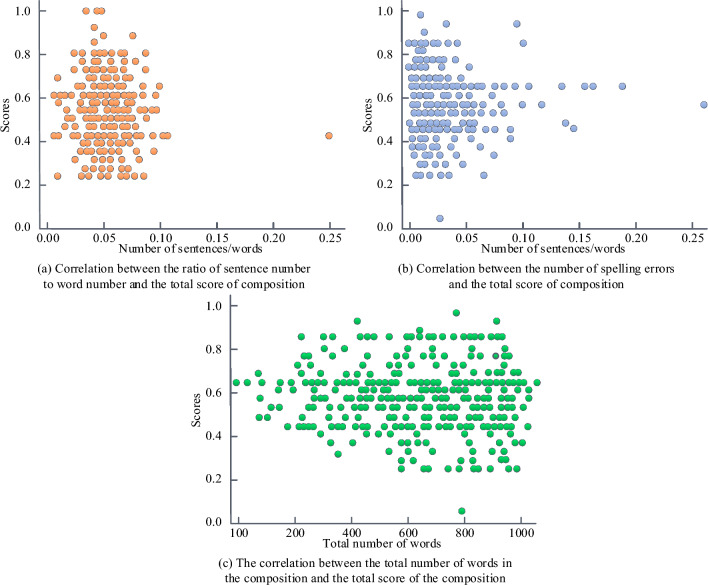
Results of correlation analysis between word characteristics and the total score of English composition.

To measure the effectiveness of feature extraction results, the study conducts a correlation analysis between word features and English composition scores. The third group of dataset P is tested. Figure 7a presents the correlation between the ratio of the number of sentences to the number of words and the total score of the composition. Figure 7b indicates the correlation between the number of spelling errors and the total score of the composition. Figure 7c displays the correlation between the total number of words and the total score of the composition. From Fig. 7, the three features had certain correlation with the composition score. However, a single feature couldn’t determine the composition total score, and the composition score presented a normal distribution as a whole. The ratio of the number of sentences to the number of words is a measure of the sentence complexity mastered by the writer. A low proportion indicates that the author has a high ability to organize long and difficult sentences. The change in word length is another measure of word mastery.

**Figure 8:**
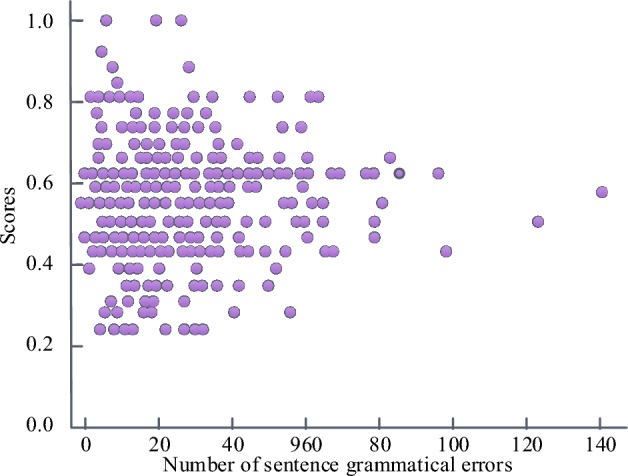
Correlation between the number of grammatical errors.

Figure 8 displays the correlation between the number of grammatical errors in sentences and the total score. The data from the third group of compositions in dataset P is selected for testing. From Fig. 8, the total score of composition presented a normal distribution, and the distribution points were relatively concentrated. They have obvious correlation between the number of grammatical errors in sentences and the score of compositions. The sentence readability metric is the weighted sum of the average number of characters in a word and the average length of a sentence. This feature can be modified in different scoring scenarios. The specific setting is 0.47*. The average number of characters per word and the average length of a sentence are − 21.43.

To study the effectiveness of the proposed DLIECG method, dataset P and the machine evaluation dataset of the correction network are used as experimental data. It is also combined with BiLSTM model, RNN model and Intelligent Scoring Algorithm for English Writing Quality Based on Machine Learning (MLIS) ^22^. The results are shown in Table 4. Among them, the training process of the RNN model is as follows. Firstly, the dynamic state of an NN is considered as a short-term memory. Secondly, a special module is created to extend the short-term memory to the long-term memory by allowing the information to be enclosed in it. Then the information is released when needed. In this process, the door is closed, so the information arriving during this period will not affect the memory state. The training process of BiLSTM model is as follows. Firstly, the English essay text is represented by an essay vector in the form of sentence granularity through the BERT pre-training model. Then it is sequentially input into the model. Secondly, two terminal output vectors of BiLSTM are extracted, which obtain the pretext information and the post-text information respectively. Then the two vectors are concatenated to get a new vector. Finally, through the full connection layer, the Sigmoid function is used to obtain the score value in the range of 0–1. The essay score of manual evaluation is based on multi-dimension consideration. Experts in each dimension will give different score values. Finally, a total score is provided, so the score of each dimension has a certain correlation with the total score value. Therefore, the evaluation method of RNN model and BiLSTM model uses Per to evaluate the correlation between the two variables: the correlation score and total essay score. Compared with BiLSTM and RNN, Table 4 presents the findings. The function of DLIECG exceeded of RNN and BiLSTM. The performance of DLIECG method on dataset P was significantly better than that of the online machine evaluation, with a maximum of 0.980.

**Table 4:** Comparison of experimental results of different methods of correcting English compositions.

Data set	DLIECG method	RNN	BiLSTM	MLIS
P-1	0.980	0.680	0.820	0.870
P-2	0.890	0.600	0.750	0.810
P-3	0.710	0.650	0.690	0.680
P-4	0.920	0.650	0.770	0.790
P-5	0.900	0.720	0.780	0.820
Correct online machine evaluation data	0.850	0.680	0.760	0.800

## Conclusion

In recent years, artificial intelligence technology has been adopted in various aspects. The automatic grading of English compositions has also received great attention. However, the representation of text content has not made much progress. To better represent the text content and build a reliable scoring system, a TRSR calculation method based on topic richness is proposed. A DLIECG feature extraction combining artificial features and deep learning is designed. From the findings, the TD achieved the best effect when it was iterated to 80 times. The accuracy, recall and F1 value were 0.97, 0.93 and 0.95 respectively. The loss value of model training began to stabilize and finally stabilized at 0.03. The accuracy and F1 value proposed in the study were the highest, at 97.83% and 95.36% respectively. Compared with RNN model, the accuracy and F1 value were 5.67% and 4.69% higher respectively. The overall performance of DLIECG was significantly higher than that of RNN and BiLSTM. The function of DLIECG method on dataset P exceeded the online machine evaluation, with a maximum of 0.980. In summary, the DLIECG method satisfies the results under the combined influence of multiple variables, confirming its effectiveness. The feasibility of this method has been demonstrated by combining the advantages of interpretability and portability based on deep learning essay scoring methods with the generalization advantages of manually designed features. However, this method still has some drawbacks. It provides general semantic information by using pre-trained models, but its output short text vector cannot be directly applied to downstream tasks. The feature extraction methods used in the research are the third-party tools, most of which are based on rules. Therefore, grammar and syntax errors cannot be effectively detected in complex and diverse English expressions. In the future, the tasks of grammar and syntax checking can be further studied.

## Data Availability

The datasets used and/or analyzed during the current study available from the corresponding author on reasonable request.
